# Epidemiology, Biodiversity, and Technological Trajectories in the Brazilian Amazon: From Malaria to COVID-19

**DOI:** 10.3389/fpubh.2021.647754

**Published:** 2021-07-13

**Authors:** Claudia T. Codeço, Ana P. Dal'Asta, Ana C. Rorato, Raquel M. Lana, Tatiana C. Neves, Cecilia S. Andreazzi, Milton Barbosa, Maria I. S. Escada, Danilo A. Fernandes, Danuzia L. Rodrigues, Izabel C. Reis, Monica Silva-Nunes, Alexandre B. Gontijo, Flavio C. Coelho, Antonio M. V. Monteiro

**Affiliations:** ^1^Programa de Computação Científica, Fundação Oswaldo Cruz, Rio de Janeiro, Brazil; ^2^Laboratório de Investigação em Sistemas Socioambientais, Instituto Nacional de Pesquisas Espaciais, Sao Jose dos Campos, Brazil; ^3^Centro de Ciência do Sistema Terrestre, Instituto Nacional de Pesquisas Espaciais, Sao Jose dos Campos, Brazil; ^4^Laboratório de Biologia e Parasitologia de Mamíferos Silvestres Reservatórios, Instituto Oswaldo Cruz, Fundação Oswaldo Cruz, Rio de Janeiro, Brazil; ^5^Ecologia Evolutiva e Biodiversidade, DGEE, Universidade Federal de Minas Gerais, Belo Horizonte, Brazil; ^6^Instituto de Ciências Sociais Aplicadas e Núcleo de Altos Estudos Amazônicos, Universidade Federal do Pará, Belem, Brazil; ^7^Instituto de Estudos em Desenvolvimento Agrário e Regional, Universidade Federal do Sul e Sudeste do Pará, Maraba, Brazil; ^8^Laboratório de Mosquitos Transmissores de Hematozoários, Instituto Oswaldo Cruz, Fundação Oswaldo Cruz, Rio de Janeiro, Brazil; ^9^Universidade Federal do Acre, Rio Branco, Brazil; ^10^Laboratório de Produtos Florestais, Serviço Florestal Brasileiro, Brasília, Brazil; ^11^Escola de Matemática Aplicada, Fundação Getúlio Vargas, Rio de Janeiro, Brazil

**Keywords:** biodiversity, Amazon, ecosystem service, technological trajectory, epidemiology, COVID-19, neglected tropical diseases

## Abstract

The Amazon biome is under severe threat due to increasing deforestation rates and loss of biodiversity and ecosystem services while sustaining a high burden of neglected tropical diseases. Approximately two thirds of this biome are located within Brazilian territory. There, socio-economic and environmental landscape transformations are linked to the regional agrarian economy dynamics, which has developed into six techno-productive trajectories (TTs). These TTs are the product of the historical interaction between Peasant and Farmer and Rancher practices, technologies and rationalities. This article investigates the distribution of the dominant Brazilian Amazon TTs and their association with environmental degradation and vulnerability to neglected tropical diseases. The goal is to provide a framework for the joint debate of the local economic, environmental and health dimensions. We calculated the dominant TT for each municipality in 2017. Peasant trajectories (TT1, TT2, and TT3) are dominant in ca. fifty percent of the Amazon territory, mostly concentrated in areas covered by continuous forest where malaria is an important morbidity and mortality cause. Cattle raising trajectories are associated with higher deforestation rates. Meanwhile, Farmer and Rancher economies are becoming dominant trajectories, comprising large scale cattle and grain production. These trajectories are associated with rapid biodiversity loss and a high prevalence of neglected tropical diseases, such as leishmaniasis, *Aedes*-borne diseases and Chagas disease. Overall, these results defy simplistic views that the dominant development trajectory for the Amazon will optimize economic, health and environmental indicators. This approach lays the groundwork for a more integrated narrative consistent with the economic history of the Brazilian Amazon.

## 1. Introduction

The Amazon basin is home to the largest tropical forest in the world, covering eight South American countries and one of France's overseas territories. The maintenance of this biome is mandatory for planetary health (1) and is invaluable to the world due to its unique biodiversity, human culture, climate regulation, gene banks and freshwater reservoirs, to name but a few social and ecosystem services (2). Approximately two thirds of the Amazon basin are located within Brazilian territory. In Brazil, there are two official boundaries for the so called Amazon region: the Legal Amazon^1^, a political-administrative definition that encompasses 58.9% (ca. 5 million km^2^) of Brazilian territory and the Amazon biome, corresponding to a biogeographic area covering ca. forty-nine percent of the country's territory (4.2 million km^2^) (3). The Legal Amazon is home to a wide diversity of cultures, languages and types of human settlements, including indigenous, quilombola and riverine communities, towns and industrialized urban centers. About 30 million people currently inhabit the Legal Amazon, ~12.5% of the total Brazilian population (4). From this total, 72.4% live in urban areas varying from small towns displaying different rurality degrees to large metropolitan regions, such as Belém and Manaus (5). In addition, 355 thousand Indigenous individuals inhabit 383 demarcated Indigenous lands (6). Forest maintenance requires understanding and caring for cultural and productive practices that seem to have established a healthy balance between direct or indirect Amazon forestry activities, having co-evolved in the Amazonian context and remained resilient until now.

Since 2012, after the lowest deforestation rate observed in three decades, a strong upward trend in Legal Amazon deforestation rates are now being witnessed, reaching 11,088 km^2^ in 2020 (7). This forest suppression is mainly driven by land demands for the implementation and expansion of new pasture areas. Large-scale agriculture also causes indirect pressure on the forest, as pastures are converted into agricultural lands. This process promotes the creation of new pasture areas by further deforestation (8–10). The rural economy of the Legal Amazon in 2018 was ca. R$ 65 billion^2^, corresponding to 12% of the region's total Gross Domestic Product (GDP). Large-scale agriculture, illegal logging and mining activities are characterized by intense conflicts during land accumulation processes, as land is one of the most valued social assets in the Amazon biome (12). Large-scale agricultural and mining projects are supported by high economic, technological and financial incentives as well as investments in large infrastructures prioritizing road building, hydroelectric dam construction, as well as freight railways and berth and bulk port terminals for commodity exports. On the other hand, rural production systems based on agroextractive and smallholder livestock activities that have persisted through the last centuries still exhibit a strong presence in the Amazon agrarian economy (13, 14). Although these sectors lack economic and fiscal incentives when compared to the agribusiness sector, they remain an important way of life for a large portion of the population that strongly relies on provisional ecosystem services and natural capital.

Deforestation and habitat fragmentation lead to several negative effects on ecosystem services, such as loss of biodiversity, soil and water quality and increased abundance of disease reservoirs and vectors in contact with human communities (15–17). Leishmaniasis, malaria, Chagas disease, leptospirosis and dengue, are all neglected tropical diseases prevalent in the Amazon region and are indicative of social and environmental vulnerability, including poverty, poor sanitation, and lack of clean water supplies.

In 2020, the vulnerability of the Amazon region to directly transmitted diseases became evident during the COVID-19 epidemic. This emergent viral disease was discovered in December 2019 in China and was declared a pandemic by the World Health Organization on 11 March 2020. On 13 March 2020, the first case was confirmed in Manaus, rapidly evolving to a large epidemic with 32259 confirmed cases and 1957 deaths in 4 months (18–20). Initially present in cities, COVID-19 rapidly spread to rural and forest communities, causing large indigenous and riverine community losses. This disease exacerbated the inequality gap and brought to light regional precarities, mainly associated with the uneven distribution of access to collective consumption goods, sanitation, and basic health services, directly impacting the living conditions of the Amazon population.

We advocate that, in order to maintain the forest and its planetary services, we must move beyond disciplinary knowledge and consider that epidemiology, economy and ecosystem services are intertwined components of the complex Amazon biome system, affecting biodiversity and the well-being of local populations. Assessments on how the state of this adaptive complex system is affected by economic development pathways, in particular, those related to the local agrarian economy, which comprises one of the main forces driving the future of the region, are paramount. We, therefore, seek to determine proper wealth, health and environmental integrity measurements that take into account the singularities of the Brazilian Amazon region. The need for new measures for wealth characterization, as well as new economic indicators concerning well-being, is now at the center of discussions regarding economic development models and policies (21–23). Using a series of indicators, we characterized the environmental and epidemiological states of municipalities following different techno-productive trajectories (TT) in the Amazon region. TT is a concept derived from a framework developed by Costa (12) and Costa (14) to model the agrarian economy of the Brazilian Amazon. This framework describes the rural reality of the Amazon region according to its structural historical-geographical diversity (13). With this approach, a more integrated and consistent narrative is produced to explain the scenarios that create or maintain ecosystems and human health in the Amazon.

In the following sections, we introduce the concept of techno-productive trajectories and describe their distribution in the Amazon. Then, we present how environmental and epidemiological indicators are associated with these trajectories forming a co-evolving system.

## 2. The Brazilian Amazon Techno-Productive Trajectories

Until the 1920's, the agricultural frontier advancing within the Brazilian Amazon established productive structures that alternated predominantly between those based on compulsory labor and those based on relatively autonomous agriculture and extractive work (13). This historical context concerning the agrarian Amazon economy is reflected today in the presence of two main microeconomic rationalities and their interactions, as follows: (i) family centrality in decision-making processes, subordinating the marginal efficiency of the capital to the logic of family and life reproduction and (ii) an economy where production essentially depends on wage labor, where economic agents behave predominantly guided by assessments concerning the marginal efficiency of the capital, i.e., oriented by profit. These two microeconomic rationalities synthesize the strategies and contexts in which economic agents make their decisions in the agrarian Amazon and are associated with the Amazon‘s Peasants and Farmer and Rancher economic agents (13, 14, 24, 25).

These two distinct rationalities are guided by two major technological solution patterns, comprising Technological Paradigms (14, 26), within different rural production systems. The Agricultural Paradigm, herein represented by intensive temporary crop systems, large scale cattle raising, large permanent crops, planted forests and technified silviculture, defines a production process based on technologies targeting the efficient mechanical, chemical and biological control of nature to achieve large-scale production. The other is the Agroextractivist Paradigm, defined from the Peasant's form of production that has persisted and evolved over the centuries, characterized by the structural diversity of their production systems, which presuppose Amazon biome diversity maintenance and coexistence.

**Figure 1:**
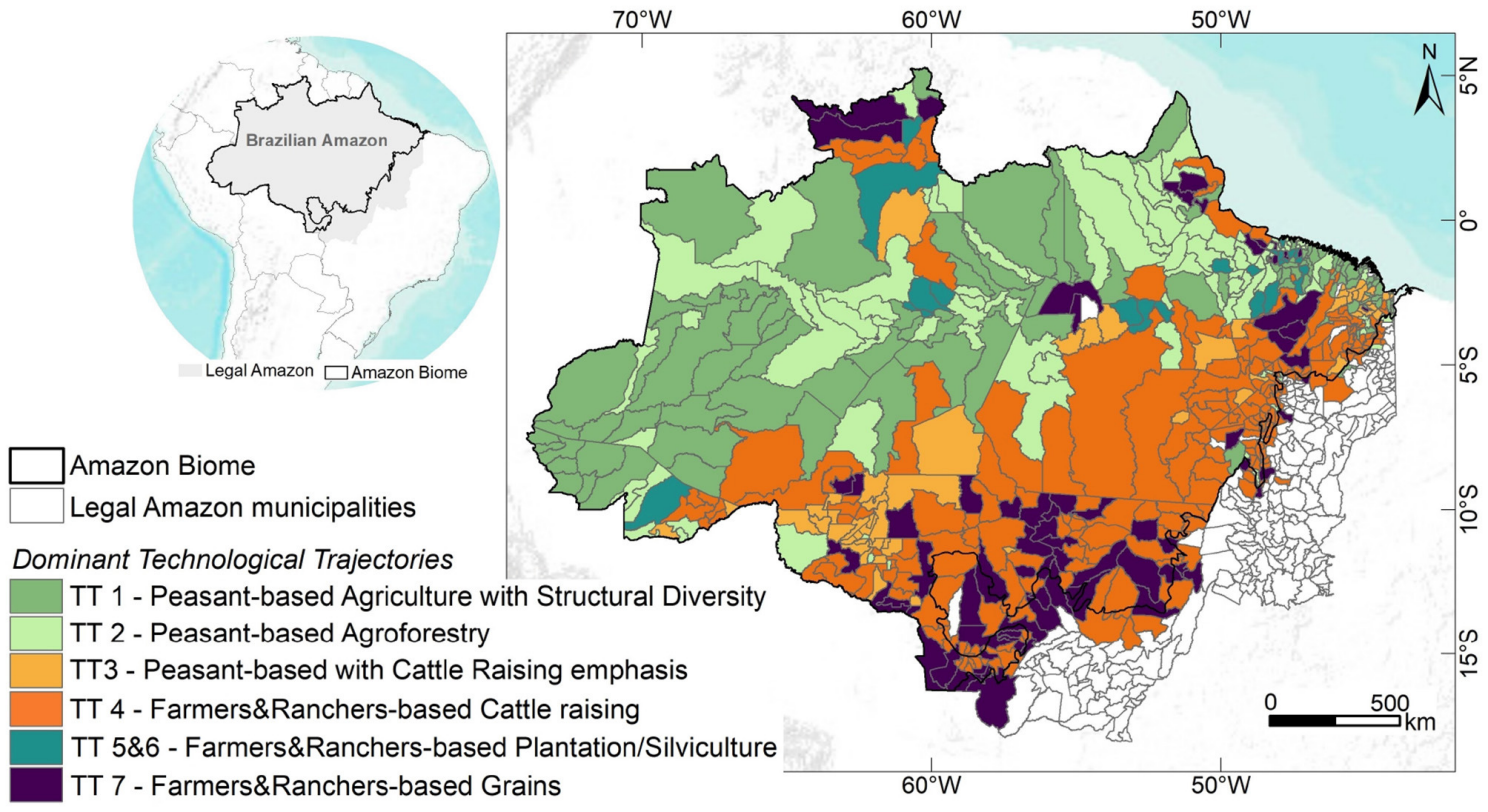
Dominant Technological Trajectories (TT) in Amazon biome municipalities in 2017. The inset highlights the limits of the Brazilian Amazon (Amazon Biome and Legal Amazon).

Techno-productive Trajectories or Technological Trajectories (TTs) emerge from the combination of these two rationality patterns and their corresponding paradigms (Agricultural and Agroextractivist) regarding the relationship between economic agents and nature, expressed in their production systems. To identify these TTs, Costa (14) developed a complete operational method consisting of four steps. The method applies multivariate regressions and principal component and factor analysis techniques to data collected by the Brazilian 1995, 2006, and 2017 agricultural censuses. Using this approach, six^3^ different technological trajectories were identified and characterized. Table 1 presents a synthetic description of these trajectories as well as the footprints they have left on the biome's landscape. We also qualitatively described each landscape footprint based on forest-nonforest spatial patterns left by economic trajectories and observed by satellite images (27). The percentage shares of the TTs in relation to the agrarian component of Amazon's gross domestic product were determined by Costa (14). Figure 1 presents a map of the dominant technological trajectories per municipality using the most recent 2017 national agrarian census data (14).

**Table 1:** Technological Trajectories and their contemporary empirical forms of expression in the Amazon biome and their associated landscape structures.

**Technological trajectories (TT)**			**Landscape footprints description**
Peasant Systems	TT1	Production systems that converge to the agriculture of permanent (cocoa, pepper, coffee) and temporary (manioc, corn, rice and beans) crops with varying compositions and diversity, but still maintaining a level of structural diversity in their operation.	*Land Mosaics with Forests*. Heterogeneous land cover mosaics composed of small temporary and permanent crops, secondary vegetation in different stages, small pasture and large continuous forest areas.
	TT2	*Agroforestry production systems*. Agroforestry production systems. Mainly comprising two types: One based on non-timber extraction (acai, nuts, waxes, rubber, oils - andiroba, copaíba, etc.) and the other based on agroforestry with permanent crops (cocoa mainly). Both are deeply rooted in structural diversity as an essential ecological context for production.	*Forest Dominant*. Predominance of large continuous forest areas, which may or may not contain small patches of secondary vegetation and permanent crops in association to the forest cover.
	TT3	Productive systems that converge to small/medium cattle ranching with the production of dairy products or beef cattle often associated with temporary (manioc, rice, beans, corn) and/or permanent crops (cocoa, peeper, coffee).	*Grassland Dominant*. Predominance of small and medium pasture areas, which may contain shrubs and trees (unmanaged pasture) associated with small cultivation areas, secondary vegetation in early stages and fragmented forests.
Farmers and Ranchers systems	TT4	Productive systems that converge almost exclusively to livestock for beef production. These systems may present crops comprising foraging species for livestock, like corn and sugarcane.	*Grassland*. Homogeneous landscapes produced by the dominance of large clean (managed) pasture areas with small patches of fragmented forests.
	TT5 and 6	Productive systems based on the cultivation of permanent crops (TT 5), such as palm oil (dendê) or upland irrigated acai, and silvicultural systems (TT 6), with the cultivation of exotic and native forest species and the extraction of products like wood, firewood, nuts, waxes and gums, among others.	*Cultivated Forest*. Homogeneous landscapes generated by the dominance of large patches containing one or few species of planted trees and shrubs. In the case of forestry, some recent wood harvest areas may occur. The landscape may or may not present forest remnants.
	TT7	Productive systems oriented to temporary crops presenting the strong use of mechanical and/or chemical technologies, primarily for grain cultivation (soybeans, rice, corn, etc.).	*Crop Landscape*. Homogeneous landscape generated by the dominance of large patches of a single crop with or without few and small forest remnants.

To calculate the dominant technological trajectory for each municipality, we computed which of the six TTs was responsible for over 50% of the municipal Gross Agricultural Product Value, that is, the total contribution value derived from the rural economy to the Municipal Gross Domestic Product in 2017. We observed that Peasant trajectories (TT1, TT2, and TT3) are dominant in ca. fifty percent of the Amazonian territory, mostly concentrated in areas covered by continuous forest. On the other hand, a strong presence of TT4, a non-peasant trajectory, linked to large cattle raising, is noted in the Southern and Eastern portion of the biome. This activity is expanding toward areas that still exhibit large amounts of forest cover. It is important to note that TT4 trajectories appear in many municipalities that also present TT3 trajectories. These two trajectories, one based on peasant rationality and the other on profit-oriented rationality, can interact through competition or cooperation. Presently, the TT4 trajectory is more likely to become the dominant trajectory in these municipalities, given current institutional arrangements. TT7 is dominant in the Southern and Northern Amazon, mainly associated with grain production, i.e., soybean and rice. Finally, non-peasant trajectories TT5 and TT6 are located at the boundaries between continuous forest and pasture.

## 3. Biodiversity Trajectories

The main biodiversity threats in the Brazilian Amazon ecosystem include deforestation and the expansion of livestock and industrialized monocultural agriculture activities over new areas. This follows a hasty industrialization process since the 1950s and, more recently, a nationwide attempt to adapt Brazil to economic globalization. In this sense, the distinct technological trajectories found across the Amazonian landscape are the primary drivers shaping the environment and its biodiversity (28).

There is unequivocal evidence that environmental change and the unsustainable use of natural resources decrease biodiversity by causing local extinctions, increasing the dominance of few species and homogenizing biotas through species introduction (29, 30). These biodiversity changes can potentially affect the occurrence of infectious diseases in humans and other taxa, including wildlife and domesticated animals (31). For instance, deforestation and habitat fragmentation increase the likelihood of contact between humans and zoonotic pathogens (15–17). This connection between environmental degradation and disease emergence has already been demonstrated for several diseases and environments (32). However, the precise mechanisms of increased disease transmission following anthropogenic environmental impacts are still poorly investigated and understood, especially in the Amazon.

The conservation status of an ecosystem is often assessed through biodiversity indicators, such as species richness and composition, endemism areas, phylogenetic composition and species conservation status (33). These metrics may correlate with the potential products and services provided by biodiversity, such as decreased or increased risks of disease (34). However, despite the Amazon's importance and huge geographic area, its biodiversity is still poorly known (35) and biodiversity data on short temporal and local spatial scales are still scarce for this region. Furthermore, biodiversity is a complex multifaceted concept that includes space and time scales and entities such as species, traits and evolutionary units (36). Thus, estimating the consequences of biodiversity loss and the erosion of ecosystem function and services on planetary health greatly depends on the considered biodiversity indicator and scales. We argue that a broad evaluation of the processes driving the structure and dynamics of biodiversity on multiple spatial and temporal scales is key to model and better understanding the ecological and evolutionary mechanisms linking landscape change to zoonotic disease emergence.

Due to a lack of better Amazon indicators, landscape degradation and deforestation are adequate proxies that may be applied to characterize the temporal and spatial environmental trajectories induced by the different uses of biodiversity and its natural resources. Peasant systems are predominantly characterized by mosaics of heterogeneous agropecuary use, secondary forest fragments and large portions of continuous forest remnants, leading to a highly diverse landscape that may sustain higher biodiversity. In contrast, Farmer and Rancher systems are dominated by homogeneous landscapes with the predominance of generalist habitat and synanthropic species, harboring lower biodiversity. The temporal dynamics of TT dominance and transitions leave landscape imprints on short and long-term time scales, and alteration patterns of these landscape footprints are used to characterize environmental trajectories.

Herein, we considered remote sensing indicators regarding vegetation cover and deforestation for each Brazilian Amazon municipality (Table 2, Supplementary Figures 1, 2, and Supplementary Table 1), in order to characterize environmental trajectories and their association with TTs. First, we computed the proportion of municipalities with original forest physiognomies and with non-forest physiognomies (savanna, grasslands and wetlands, among others), as the Legal Amazon presents other physiognomies besides the tropical rainforest. Second, using deforestation data (7), we computed the percentages of remaining forest area until 2017 (Remn forest), deforestation from 2006 to 2017 (Def 2006–2017) and the percentage of the total deforested area until 2017 (Def by 2017) for each municipality. A detailed description of these indicators is found in the Supplementary Table 1.

**Table 2:** The values correspond to the percentage (%) of municipalities following a techno-productive trajectory classified as presentng “high values”.

	**Proportion of municipalities with high values**					
**Environmental descriptors**	**TT1**	**TT2**	**TT3**	**TT4**	**TT5&6**	**TT7**
**Original phytophysiognomy**						
Forest physiognomy	66.0	50.0	75.0	72.0	81.0	47.0
Non-forest physiognomy	1.0	5.2	0.0	7.6	4.8	20.3
**Habitat and Habitat loss**						
Deforestation 2006 - 2017	21.0	20.8	39.6	40.6	33.3	47.3
Deforested area up to 2017	25.0	13.0	38.0	41.0	24.0	16.0
Forest remnants in 2017	47.0	41.7	22.6	18.3	38.1	20.3
**Diseases**						
**Environmental borne**						
Hantavirus (2009-2013)	0.0	0.0	0.0	2.5	0.0	6.8
Schistosomiasis (2010-2014)	4.5	0.0	13.2	2.5	0.0	4.1
Leptospirosis (2013-2017)	10.0	16.7	15.1	6.6	9.5	5.4
**Vector borne**						
Spotted fever (2008-2013)	0.0	0.0	0.0	0.5	0.0	0.0
Chagas disease (2014-2018)	7.3	14.6	0.0	0.0	9.5	0.0
Visceral Leishmaniasis (2014-2018)	5.5	8.3	3.8	18.3	4.8	8.1
Malaria (2014-2018)	39.1	45.8	24.5	14.2	28.6	8.1
American cutaneous Leishmaniasis (2014-2018)		28.1	30.2		28.6	32.4
Aedes-borne diseases: dengue, Zika and chikungunya (2014-2018)	9.1	11.5	24.5	31.5	14.3	50.0
**COVID-19** (2020)	25.0	37.5	3.8	22.4	33.3	24.0

*A “High value” is defined as belonging to the top quartile of the frequency distribution. Color intensity is illustrative for ease of viewing and is proportional to the values as represented on the scale*.

Forest conversion is considered an important biological change driver and a meaningful proxy for habitat loss (37). Recent studies have demonstrated the importance of habitat amount (38), landscape and within-forest disturbances (39), and landscape configuration (40) to explain biodiversity declines following deforestation. A survey of multiple agricultural areas (landscape scale) in the Amazon indicated that overall local biodiversity dropped steeply when forest cover fell below 30–40% and when forest patches reached 50% of undisturbed forest (41). Studies also underline the importance of old secondary vegetation, managed forests, and tree plantations in the maintenance of local species richness for different groups of plants and animals (39, 42).

## 4. Epidemiological Trajectories

The Twentieth century is characterized by an overall transition from infectious to chronic diseases as the main causes of death in several countries. This epidemiological transition is attributed to the discovery of etiological agents and transmission cycles, city sanitization and more effective prevention and health promotion strategies, as well as more effective treatments. Many diseases have been eliminated or controlled, such as measles, polio and tuberculosis, among others (43). Meanwhile, we are witnessing the emergence and reemergence of new infectious diseases triggered by demographics, transportation and environmental changes.

In Brazil, life expectancy improvements and decreased death rates by communicable diseases, especially diarrhea, lower respiratory infections, tuberculosis, meningitis, and vaccine-preventable diseases are noted (44). However, compared to other Brazilian regions, the Amazon region has maintained the worst health indicators. The median age at death was 60 years in 2008 and remained the same until 2013, while other Brazil regions gained at least 5 years of life. Neglected tropical diseases are an important morbidity and death cause in the Amazon, and the median age of death by infectious diseases was 50 years old in 2013 (45). This region also displays the highest infant mortality rate in the country (21.8 deaths per 1,000 births) and the second lowest life expectancy at birth (72.43 years) (46).

Neglected tropical diseases (NTDs) are infectious diseases presenting chronic and debilitating characteristics, prevalent in low-income countries and more concentrated in extremely poor populations (47). Poor housing and working conditions and a lack of access to preventive health services and assistance are social determinants for these diseases. Many NTDs are zoonotic diseases, and their dynamics also depend on environmental determinants, such as regulating and supporting ecosystem services (48). Herein, we collected data on zoonotic diseases reported to the Brazilian Ministry of Health (see details in the Supplementary Material) and analyzed their distribution among municipalities following different technological trajectories. The data comprise vector-borne NTDs (dengue + Zika + chikungunya, Chagas disease, visceral and cutaneous leishmaniasis, vivax malaria) and non-NTDs (spotted fever) as well as diseases directly associated with environmental degradation, including rodent- and water-borne diseases (leptospirosis, hantavirosis and schistosomiasis). These diseases follow a spectrum of urban to rural diseases, with varying degrees of association with biodiversity, land use and land cover. Finally, we also analyzed the spatial distribution of COVID-19 that invaded the Amazon region on March 13th 2020 and spread quickly into a large epidemic.

We calculated the accumulated incidence for each disease in a time window of 5 years (Supplementary Table 1). The specific time window varied to accommodate data availability differences. The population in 2015 was used as the denominator. For COVID-19, we calculated the accumulated incidence in 2020, using surveillance data collected up to April 1st 2021. The estimated population in 2019 was the denominator. Municipalities within the top 25% of accumulated incidence were classified as “high risk.” This indicator is robust when applied to data varying from highly prevalent endemic diseases to more focal diseases with episodic outbreaks.

### 4.1. Vector Borne Diseases (VBD)

**Figure 2:**
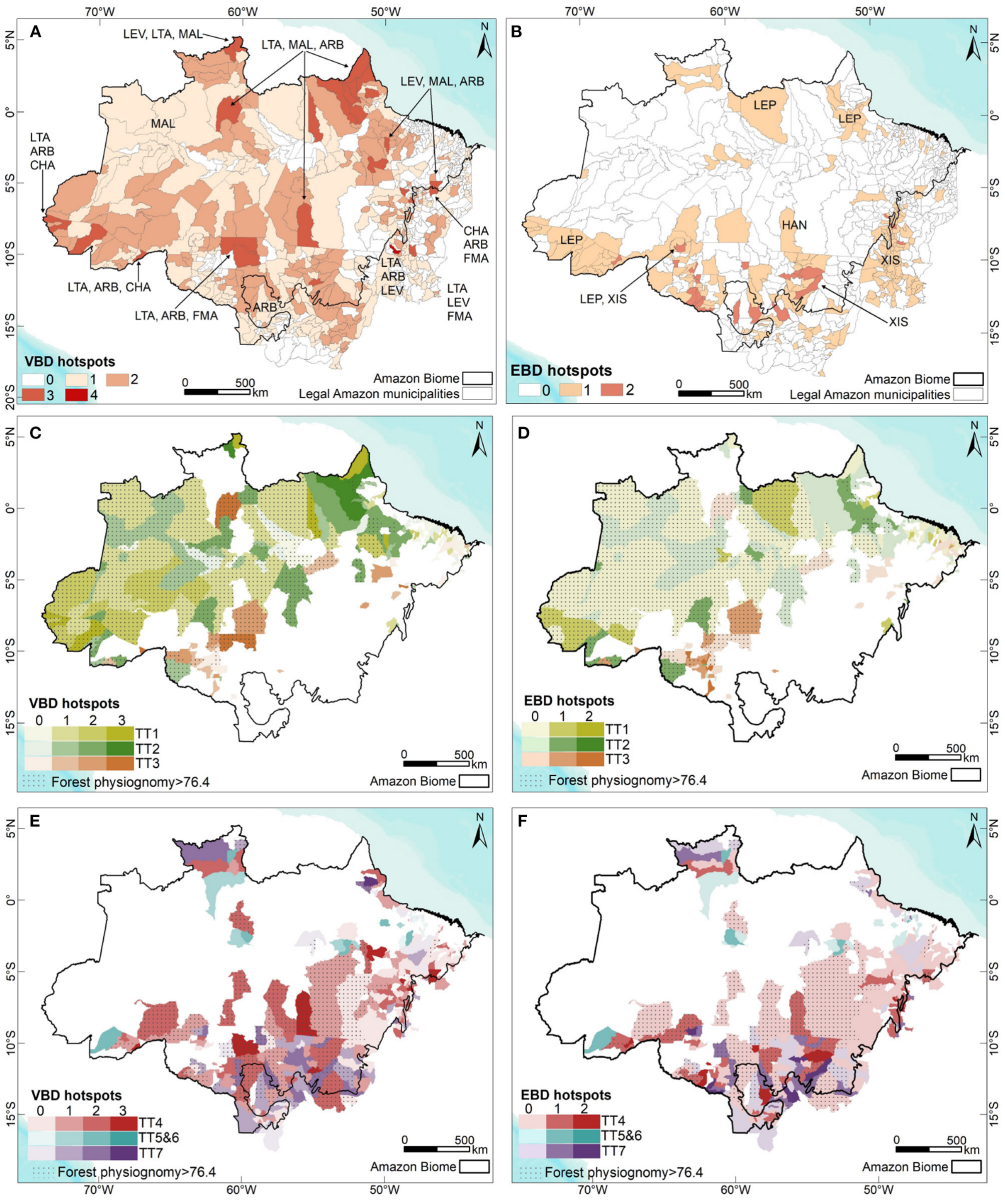
**(A)** Municipalities presenting the occurrence and co-occurrence of Vector-borne diseases (VBD); **(B)** Municipalities presenting the occurrence and co-occurrence of schistosomiasis, hantavirosis and leptospirosis (EBD); Municipalities dominated by Peasant Trajectories (colors), forest physiognomy (hatch) and the occurrence of VBD **(C)** or EBD **(D)** (color intensity); Municipalities dominated by Farmer and Rancher Trajectories (colors), forest physiognomy and occurrence of VBD **(E)** or **(F)** EBD (color intensity).

Supplementary Figure 3 displays maps concerning the accumulated incidence of Aedes-borne diseases (dengue + Zika + chikungunya), american and visceral leishmaniasis, Chagas disease, and spotted fever in the Brazilian Amazon. A map of the annual parasite index (API) for malaria is also shown. Figure 2A displays the municipalities where one or more of these VBDs co-occur at higher sintensities.

**Malaria (MAL)** is still an important cause of years of life lost to disability, particularly in children and young adults (49). It is also associated with preterm birth and low birth weight in women lacking access to prenatal care (50). Most malaria cases in the Amazon are caused by *Plasmodium vivax*, an NTD (51, 52). Malaria vectors breed in shaded clean and still water, like lakes, the borders of rivers and streams, and small transient puddles formed in flooded forests. Roads and canals that create artificial pools, as well as fish tanks close to flooded forests, are examples of human constructions that may amplify mosquito populations (53). *Anopheles darlingi*, the most important malaria vector in the region, has adapted well to these artificial environments but other *Anopheles* species displaying different vector competence degrees and habitat preferences are also found in the region (54–57).

**Aedes-borne diseases (ABD)**. The Brazilian Amazon was the port-of-entry of DENV-2 in 1982 (58), DENV-4 in 2010 (59) and chikungunya in 2014 (60). Urban centers in the Amazon suffer from poor garbage collection services and piped water. These factors create environmental conditions that facilitate the maintenance of a high abundance of *Aedes* spp. Approximately 58 thousand cases are reported each year, mostly dengue (76%), followed by chikungunya (15%) and Zika (9%). Other ABD, such as Marburg, although detected, are not monitored by routine epidemiological surveillance efforts.

**American cutaneous leishmaniasis (LTA) and visceral leishmaniasis (VL)** are diseases caused by protozoans belonging to the *Lutzomyia genus*. Sandfly vectors are abundant in humid forests (61) but have adapted to secondary forests, tree plantations and green spaces in rural and urban areas (62). In the past, LTA was a major cause of illness in extractivist communities, alongside malaria. As ruralization and urbanization progressed, the LTA transmission cycle also adapted which is evident in the homogeneous distribution of this disease along all TTs (Supplementary Material). An average of 7,000–11,000 LTA cases are reported per year. Although its displays low lethality, this neglected tropical disease is a cause of social stigma. Cure depends on aggressive treatment since spontaneous cure occurs in only 6% of all cases (63). The ecological plasticity of LTA is explained by the diversity of potential vertebrate hosts, including both wild and domestic *Canidae*, rodents, and marsupials, as well as a vector adaptation to feed on humans and peridomestic animals (64). In the Amazon, new leishmaniasis foci have been associated with deforestation followed by farming (65). VL, the visceral form of leishmaniasis is more concentrated on the eastern part of the Amazon and north of Roraima, in the transition region between the forest and non-forest biomes. From 900 to 1,500 cases on average are reported each year, with a lethality rate ranging from 5 to 7%.

**Chagas Disease (CHA)** is an endemic disease with an enzootic cycle involving wild mammals (Marsupialia, Chiroptera, Rodentia, Edentata, Carnivora and Primata) and forest-dwelling triatomine vectors. Two to three hundred new cases are reported each year. Higher incidence areas are concentrated in Pará, around the city of Belém, and in the state of Acre. Oral transmission is also detected, associated to the consumption of açaí and other palm fruits.

**Spotted fever** is a bacterial disease caused by the *Rickettsia* genus, usually transmitted by ticks. In Brazil, most cases are reported in the Southeast region, with capybaras and horses as the main animal reservoirs. Although not endemic in the Amazon region, 10–20 cases have been reported each year in the transition area in Tocantins and Mato Grosso states. Diseases caused by *Rickettsia* spp. are likely to be highly under-diagnosed in the Amazon region, in part due to the lack of awareness (66). Recently, the disease was described as being caused by *Rickettsia typhi* in the Amazon, transmitted to humans by fleas. In 2009, a rickettsiosis outbreak was investigated in an indigenous population in the state of Mato Grosso (67). Better tools for monitoring rickettsioses should, therefore, be a priority in the Amazon.

### 4.2. Other Environmentally Borne Diseases (EBD)

Supplementary Figure 4 presents accumulated incidence maps for leptospirosis, hantavirus and schistosomiasis. Figure 2B displays the municipalities where one or more of these EBDs co-occur at higher intensity.

**Leptospirosis (LEP)** is an acute febrile illness caused by bacteria belonging to the *Leptospira* genus, transmitted to humans through contact with water contaminated with urine from infected rodents. *Leptospira* can remain viable in water for several months (68) and is considered a doubly neglected disease due to the lack of awareness of the Brazilian population regarding its severity (69). Endemicity is associated to urban areas with poor sanitation and open sewers or rural areas where agricultural practices lead to water contamination with animal urine. In agricultural settings, pigs and cattle can also act as reservoirs for *Leptospira*. Large leptospirosis outbreaks often occur after flooding events, common during the heavy rain months in the Amazon. For example, a molecular study carried out in the Peruvian Amazon reported heavy contamination of river water with rat urine (70). Cases are likely highly under-reported due to difficulties concerning Leptospirosis diagnoses.

**Hantavirus infections (HAN)** comprise zoonotic infections that have wild rodents as reservoirs. In the Americas, hantaviruses cause Hantavirus Pulmonary Syndrome (SPH). Human infection occurs through the inhalation of secretions or excreta from wild and synanthropic rodents from different species, predominantly in grain production settings that concentrate a large density of rodents. In the Amazon, the number of reported hantavirus infection cases is small compared to other areas in Brazil, concentrated in Mato Grosso and Southern Pará (71, 72). On the other hand, a serological survey in municipalities with forest economies (73) reported a low prevalence of hantavirus infections. Studies have demonstrated that the transmission of hantavirus is sensitive to biodiversity, although specific mechanisms may differ between places (74).

**Schistosomiasis (XIS)** is a helminthic disease caused by *Schistosoma mansoni*, whose intermediary hosts are aquatic snails belonging to the *Biomphalaria* genus. The transmission cycle involves contamination of snail-inhabiting lakes by infected human feces. The receptivity of the Amazonian limnological environment to the introduction of *S. mansoni*, and the risk posed by the arrival of migrants from endemic areas of the country to work in rubber plantations was already known in the 1950's (75). XIS is found in higher prevalence in municipalities located in the southern border of the Amazon (Table 2). These areas have attracted immigrants from endemic regions that end up inhabiting areas with poor sanitation infrastructure where the XIS transmission cycle has a high probability of becoming endemic (76). There is evidence that the acidic water in part of the Amazon region has acted as a barrier against XIS expansion, although, more studies are required to identify other hosts that may participate in the transmission of this disease in the region (77).

### 4.3. COVID-19

Supplementary Figure 5 displays the accumulated incidence of COVID-19 in the Brazilian Amazon during 2020. This period encompasses the first epidemic wave and the inter-epidemic period, with 1.2 million cases reported, 26,349 confirmed deaths, and a lethality rate of 2.1%. In the absence of measures to reduce mobility and increase social distancing, the disease spread at full speed. The health system collapsed in April in the large city of Manaus (78). Several municipalities were intensely affected (Table 2). COVID-19 also moved very quickly into the forest, brought by chains of contacts involving health and social assistants coming from the cities or by the flow of forest dwellers fleeing from towns back home. Entire communities were hit at once (79). Supplementary Figure 5 indicates the ubiquity of COVID in this region.

## 5. Interactions Among Economic, Environmental and Epidemiological Trajectories

**Figure 3:**
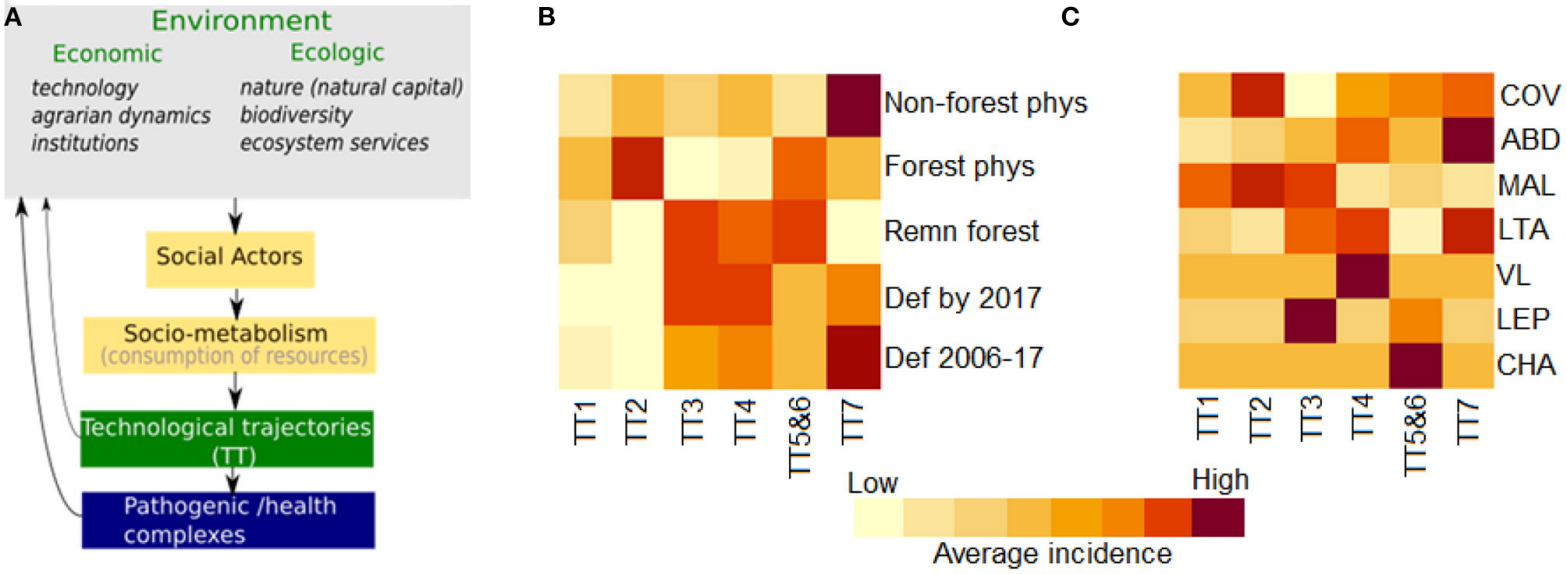
Theoretical model for the system comprising the technological, ecological and epidemiological trajectories in the Amazon region. **(A)** Diagram presenting links between economic and ecological context variables and pathogenic/health complexes mediated by the technological trajectories. **(B)** Heatmap of the median environmental and disease indicators in municipalities following different technological trajectories (see Table 1 for trajectory description, TT-1 to TT-7). Ecological indicators: recent deforestation (def 2006-2017); total deforestion prior to 2017 (Def by 2017); amount of forest remnant areas in 2017) (remn forest); forest physiognomy as the original biome (forest phys); non-forest physiognomy-savanah, rocks, natural grassland and wetland (non-forest phys). **(C)** Median disease incidence in municipalities following the assessed technological trajectories: schistosomiasis (SCH), leptospirosis (LEP), Chagas disease (CHA), visceral leishmaniasis (VLE), malaria (MAL), american cutaneous leishmasis (ACL), *Aedes*-borne diseases (ARB), COVID-19 (COV).

Figure 3A synthesizes the conceptual framework applied herein. We depart from the perspective that the changing land use and land cover mosaics observed in the Brazilian Amazon landscape are driven by the local agrarian economic dynamics. This process can be described in ecological and socio-economic terms. From a socio-economic perspective, this dynamic is well characterized by Techno-productive Trajectories (TTs). Different TTs can coexist and interact via competition or cooperation strategies, determining changes in the forested landscape. The specific relationship between production and nature in each setting will vary depending on the producers' logic, knowledge and technology, which may or may not incorporate an ecological context in their processes. Concerning the landscape, this is seen as loss of forested areas with a direct impact on habitat loss. Habitat loss is associated with biodiversity impacts (80, 81). As the natural environment is anthropized, landscape transformations create conditions for the (re)emergence of diseases and persistence of endemic cycles with varying degrees of dependence on the sylvatic environment and TT predominance (Figures 2C–F).

Figures 3B,C present heatmaps with colors proportional to the median value of each environmental indicator and disease in the assessed municipalities classified by TT type. Table 2 presents the percentage of municipalities with top values for each indicator according to TT type. Together, these indices illustrate the associations between economic and ecological trajectories in the Amazon region and the burden of selected diseases. Among the municipalities dominated by Peasant economies, those with TT2 trajectories are most concentrated in originally forested regions that underwent more intense land conversion prior to 2006, indicating older colonizations. Agroforest activities are an important component of this economy, which may explain the lower rate of recent change. Higher deforestation rates are observed more recently in TT3-dominated municipalities, which are found in both forest and non-forest physiognomies. A historical association is noted between Peasant TT3 and Farmer and Rancher TT4 trajectories with strategies of cooperation or assimilation of the trajectory TT3 by the TT4 (82). During the 2006-2017 period, municipalities dominated by TT3 and TT4 ranked in first places concerning deforestation rates. This intense conversion of forested landscapes into grassland formations followed by the establishment of cattle ranching and other agriculture activities has impacted the amount of available forest habitat, leading to biodiversity consequences. In this context, deforestation is associated with conflicts and pressure from non-peasant economies. Meanwhile, municipalities where TT1 dominates maintain larger forest remnants and lower change rates.

The distribution of vector-borne diseases among TT1, TT2, and TT3 trajectories is heterogeneous (Figure 2C). Malaria is the main cause of disease burden in all three, although more intense in TT2. Individual risk factors include working within or close to the forest, living at the border of the forest, being an immigrant from a non-endemic area, living in houses made of wood and lacking nets and scarce access to treatment (52, 83). American cutaneous leishmaniasis is concentrated in TT3 (and TT4) municipalities, characterized by the presence of large livestock herds. Chagas disease has a low median incidence in Peasant-dominated municipalities. However, some TT2 and TT1 municipalities are also located within the most affected areas (Table 2). Exposure to wild triatomines attracted by light or peridomestic animal blood meals are risk factors for forest dwellers living in TT1 municipalities. A second scenario is related to palm extractivism, such as acai and piassava, where workers are bitten by triatomines that live in the palm leaves in both TT1 and TT2 (84). A total of 24.5% of the municipalities displaying TT3 also presents a high burden of dengue and chikungunya. These municipalities are mostly located in non-forested areas in the southern border of the Amazon region, in the transition are between the forest and cerrado biomes. Higher urbanization rates in this region can explain the presence of dengue in this landscape.

Municipalities dominated by Farmer and Rancher trajectories present high deforestation rates. One-quarter of the TT5- and TT6-dominated municipalities are among the municipalities with the highest percentages of deforested areas by 2017. Forest conversion in these municipalities is often performed by the substitution of the original forest by forest plantations. The newly planted forests are merged with the forest remnants areas, but the ecosystem is ecologically different, characterized by lower biodiversity, among others.

Of all trajectories, TT7 presents the highest number of municipalities displaying Aedes-borne diseases and American cutaneous leishmaniasis (Figure 3). These municipalities, located in areas with original non-forest physiognomy, were the first to cultivate grains in the Brazilian Amazon, expanding into the forested areas after the 2000s (85). Despite the fact that most non-forest physiognomy areas are located in municipalities associated with dominant TT7 trajectories, 47% contained originally over 78% of forest physiognomy (Table 2). From 2006 to 2017, municipalities with dominant TT7 trajectories presented the highest contribution to deforestation in the Legal Amazon, followed by TT4 and TT3 (Table 2). Regarding TT5 and TT6, acai monocultures are associated with reduced biodiversity and increased abundance of parasite-amplifying hosts such as marsupials (86). The high incidence of Chagas disease in TT5 and TT6 may be associated to the consumption of uncooked foods, like acai, contaminated by the feces and urine of wild triatomines (87).

Municipalities presenting high incidences of leptospirosis are observed in all technological trajectories (Table 2), from forest to urban, particularly in areas susceptible to flooding, such as the states of Acre and Pará (88, 89). Despite this overall distribution, the leptospirosis burden is higher in TT3 and TT5 and TT6. TT5 and TT6 municipalities also exhibit high malaria incidence. These areas display relatively less forest cover, where malaria is likely associated to specific rural activities. For example, (90) found a strong association between acai production and increased malaria incidence by *P. falciparum*, higher than associations to nut extraction and agricultural activities.

## 6. Limitations of Available Metrics and Indicators

Despite its importance and huge geographic area, the Brazilian Amazon biodiversity is still poorly known (35). Recent studies demonstrate that biodiversity distribution is highly heterogeneous at both local and regional scales. A lack of studies on ecological interactions involved in the control of vector and reservoir species, as well as in pathogen virulence is noted. Adding to the challenge, the complex ecological interactions related to disease transmission and their interplay with other variables (e.g., landscape, economy, demography) form a complex system that defies causal relationships. This highlights the urgent need for understanding biodiversity dynamics and ecosystem functioning in the rapidly changing Amazon landscape.

Deforestation and forest fragmentation have already been applied as proxy indicators for habitat loss in studies addressing the relationship between environmental degradation and human health in the Amazon. A strong positive correlation between the number of malaria cases, deforestation and forest degradation in the Brazilian Amazon forest frontier has been reported, for example (91). The expansion of techno-productive trajectories linked to more intensive land uses (large areas for cattle raising and intensive agriculture) in the Amazon has resulted in an intense loss of forest habitat. However, the identification of biodiversity metrics that reflect anthropogenic disturbances relevant for epidemiology remains a challenge. Many of the metrics commonly applied to quantify biodiversity do not necessarily directly reflect the ecosystem service of disease regulation. For instance, species richness and abundance, the most basic biodiversity measures, naturally vary among distinct environments, and are not necessarily able to account for the regulatory role that ecosystems play in parasite transmission cycles. Another important biodiversity indicator used in ecological studies is endemism, although the relationship between endemic patterns and their potential contribution to the amplification or dilution of parasite transmission is not yet clear. In a local study, (92) reported that a reduced biodiversity of mammalian reservoirs led to increased *Trypanosoma cruzi* infection rates in domestic animals. This indicates that the identity of host species or even local trait distribution may better measure ecosystem functions played by certain species. This is noteworthy, as traits related to the epidemiology of parasite-host interactions determine the potential of ecological communities to amplify or dilute parasite transmission (31).

Although an increasing availability of global biodiversity data is observed, the Amazon is still poorly represented, with vast knowledge and sampling gaps. The global impacts of the COVID-19 pandemic brought forth the need to understand the direct effects of biodiversity changes on disease risk in the Brazilian Amazon. To address such a challenge, broad-scale studies aiming to describe biodiversity patterns and understand how they correlate with ecosystem services are required. Further studies in the biodiversity and health interface with the aim of surveying and monitoring the dynamics of infection rates in vectors and reservoirs are also paramount.

Furthermore, public health data limitations are also noted, as only a small set of diseases comprise mandatory notification and the surveillance system is not tailored for detecting new diseases. By measuring some separate diseases at a time and relying on clinical criteria for disease classification, a low sensitivity and low specificity surveillance system is established. This issue must be handled in order to study the association between disease and biodiversity. Moreover, incidence counts do not provide sufficient information.

Peasant trajectories with lower biome impacts, although still very present, are losing strength in the Amazon. These economies are invisible to standard economic indicators, despite the fact that they effectively contribute to the composition of the municipal GDP and are spatially distributed throughout the biome. The economic development agenda for this biome has supported and favored technological trajectories linked to the Agricultural paradigm (TT4, TT5, TT6, and TT7). The expansion of these trajectories into areas where Peasant trajectories are still strongly present is of concern (Supplementary Figures 6A,B). The fact that these regions comprise the largest continuous forest cover areas must be acknowledged. In particular, the spatial distribution of municipalities with dominant TT3 and TT4 trajectories is of special concern, as these trajectories are associated with cattle raising, one of the main deforestation-causing activities (Supplementary Figure 6C). To reach an inclusive, socially just and environmentally responsible development agenda for the Amazon, the real economy associated with the Peasant trajectories cannot be forgotten in the debate. The choices that will be made in this field will be decisive for the complex interactions between forest cover, biodiversity and disease development and emergence. We defend that novel economic indicators are required, because either the standard economic indicators contain problems and must be changed, or we will have to choose between saving economic indicators or saving the forest and the people who live in it.

## 7. Conclusion

This study groups economic, environmental and life health dimensions in the Brazilian Amazon. We demonstrate herein how environmental and health indicators differ among different technological trajectories, creating specific environmental and disease landscapes. While some diseases, like malaria and dengue, are dependent on specific socio-biodiverse complexes, this paper demonstrates that other diseases associated with specific TTs, such as LVA, have evolved to prevail in all TTs. As NTDs, these diseases comprise social and environmental vulnerability markers, and tracking these associations in other spatial and temporal scales, as well as other diseases and health outcomes, are paramount to validate this approach.

The ultimate goal of the planetary health initiative is the development of an ecosystem-human health index, combining biodiversity alteration, demographic and health and economic indicator patterns and how they change in response to different economic and social contexts. Some global indices have been proposed in the literature, such as a measure of global biodiversity intactness index by combining observational data regarding species richness and abundance, land use and land cover maps and human density maps (93), which should be properly assessed at the local and regional levels. Testing and validating or adapting these indices to local realities and devising new methodologies to adequately integrate them with health and economic dimensions is an urgent task. Understanding the role of biodiversity in regulating ecosystem services is paramount to reconstruct the barriers concerning the transfer of diseases from animals to humans in degraded environments (94). In this sense, it is crucial to consider the interdependence of ecosystem integrity and the strategies and policies deployed to develop local and regional economies. Land use and its impacts on Brazilian Amazon biodiversity will be determined by the outcome of the disputes among the different TTs present in the region. The local peoples resistance and resilient structures and production systems, although invisible by the conventional indicators, are an important part of the regional economy.

Health and well-being are not simply external environment outputs, but are strongly dependent on adaptation to local environments. Human culture, technology, genetics and physiology are aspects of this adaptation. While in the temperate zone many adaptations were required to avoid the cold and food scarcity during the winter, tropical forest dwellers evolved adaptations to support seasonal floods, heavy rains and rapid rotting. It is imperative that we abandon the notion of the forest as inhospitable for humans. What is inhospitable for one, is home for another. Solutions are local and diverse and must be acknowledged by adequate metrics. As an Amazonian poet once sang “*I don't want to be global, I want to be local*” (Eliakin Rufino).

## Data Availability Statement

Data is shared in the Zenodo repository. Access: DOI 10.5281/zenodo.5038657.

## Author Contributions

AD, AR, and ME organized the environmental dataset, while AD, RL, TN, and IR organized the epidemiological dataset. AM and DF organized the technological trajectories dataset. Maps were created by AD, AR, and IR. MB proposed the conceptual diagram. This synthesis resulted from a series of group discussions with all the authors. All authors contributed to the conception of the study and writing the final version.

## Conflict of Interest

The authors declare that the research was conducted in the absence of any commercial or financial relationships that could be construed as a potential conflict of interest.

## Footnotes

^1^The Legal Amazon or the Brazilian Amazon is the administrative area operated by the Superintendence of Amazon Development (SUDAM), created in 2007. The Amazon biome includes the Amazon Rainforest formations and associated ecosystems subject to the Rain Forest Protection Law 11.428, 2006.

^2^All figures are corrected for 2018 Brazilian Real values. Based on an average commercial exchange rate of the Brazilian-Real/US-Dollar in 2019 of 3.6542 (11), the values for the rural Gross Production Value (GPV) in US$ was of about US$ 18 billion.

^3^As the Technological Trajectory associated to the silviculture systems run by non-peasant agents, (TT6) only displays a small and local contribution to the regional agrarian economy, it was combined with TT5 (TT5 and TT6). It is important to note that TT5 and TT6 merge two distinct non-peasant TTs. TT5 consists of permanent crops (for example, palm oil) and specific agroforestry systems (such as Açaí palm with pepper) while TT6 is associated with silviculture systems.
