# Supplementary material for: Epidemiology, Biodiversity, and Technological Trajectories in the Brazilian Amazon: From Malaria to COVID-19
Source: Front Public Health. 2021 Jul 13;9:647754. doi: 10.3389/fpubh.2021.647754 (PMC8314010; doi:10.3389/fpubh.2021.647754)
Supplement: Supplementary file 1 [file Data_Sheet_1.PDF]

Supplementary material to Epidemiology, biodiversity and technological trajectories in the Amazon: from malaria to COVID-19

Article for Frontiers in Public Health: issue on Planetary Health

Here, we present more information regarding the data used in the analysis, and provide additional maps and statistics on each dimension of the Amazonian system (environmental, economy and epidemiology)

Data

Table S1 presents the environmental and epidemiological descriptors and the source of data used for their construction. Habitat loss was measured using land cover data from Prodes (INPE). Measures of forest area per municipality in 2006 and 2017, and original forest area were used to compute the proportion of forest habitat remaining in the forest area of the municipalities in 2017 and the contribution of each municipality for the total forest habitat loss in the Legal Amazon region, in the 2006 - 2017 period. The fourth quartile of the frequency distributions of these indicators was used to identify municipalities at the top rank of these environmental descriptors. The Amazon biome has other physiognomies besides the tropical forest, such as savana, cerrado, and water bodies**.** Two indicators were constructed: the proportion of the municipality with originally forested, and the proportion of the municipality with non-forest physiognomies, mostly cerrado and water surfaces. Disease data was obtained from the Brazilian Disease Notification system through the File Transfer Protocol Service (FTP link ftp.datasus.gov.br/dissemin/publicos/SINAN), and the Brazilian Official Malaria Notification System (SIVEP-Malaria).

Table S1. List of environmental end epidemiological indicators. The spatial unit is municipality.

**Indicators**	**Description**	**Threshold**	**Source**
**Habitat and Habitat loss**			
% Forest remnants in 2017	Percentage of the original forest physiognomy in 2017	57.4%	Prodes, INPE
% deforestation 2006 – 2017	Deforested area in the period divided by the total deforested area in the whole Legal Amazon area	0.08%	Prodes, INPE
% deforested area up to 2017	Total deforested area until 2017 divided by the original forest physiognomy area	79.9%	Prodes, INPE
**Original physiognomy**			
% forest physiognomy	Area originally with forest physiognomy divided by total area	78.6%	Prodes, INPE
% non-forest physiognomy	Area originally with non-forest physiognomy such as cerrado, natural grassland, stones, etc.	61.6%	Prodes, INPE
**Vector-borne diseases (VBD)**			
Malaria parasite index in 2014-2018	Number of positive malaria exams in the period / population in 2015 * 1000	11.9	SIVEP
Accumulated incidence of arboviroses in 2014-2018	Number of cases of dengue, chikungunya and zika reported in the period/population in 2015 * 1000	10.7	SINAN
Accumulated incidence of American	Number of cases reported in the period/population in 2015	3.25	SINAN

cutaneous Leishmaniasis in 2014-2018	* 1000		
Accumulated incidence of Visceral Leishmaniasis in 2014-2018	Number of cases reported in the period/population in 2015 * 1000	0.75	SINAN
Accumulated incidence of Chagas disease in 2014-2018	Number of cases reported in the period/population in 2015 * 1000	0.19	SINAN
Accumulated incidence of Spotted fever in 2008-2013	Number of cases reported in the period/population in 2015 * 1000	0.21	SINAN
**Environment-borne diseases (EBD)**			
Accumulated incidence of leptospirosis in 2013-2017	Number of cases reported in the period/population in 2015 * 1000	0.19	SINAN
Accumulated incidence of Hantavirus in 2009-2013	Number of cases reported in the period/population in 2015 * 1000	0.16	SINAN
Accumulated incidence of Schistosomiasis in 2010-2014	Number of cases reported in the period/population in 2015 * 1000	0.20	SINAN
**COVID-19**	Number of cases reported until week 43-2020/population in 2015 * 1000	41.6	Brasil.io[1]

[1] https://brasil.io/

Figure S1 shows the 5 years accumulated incidence of 6 vector-borne diseases in the Amazon region.

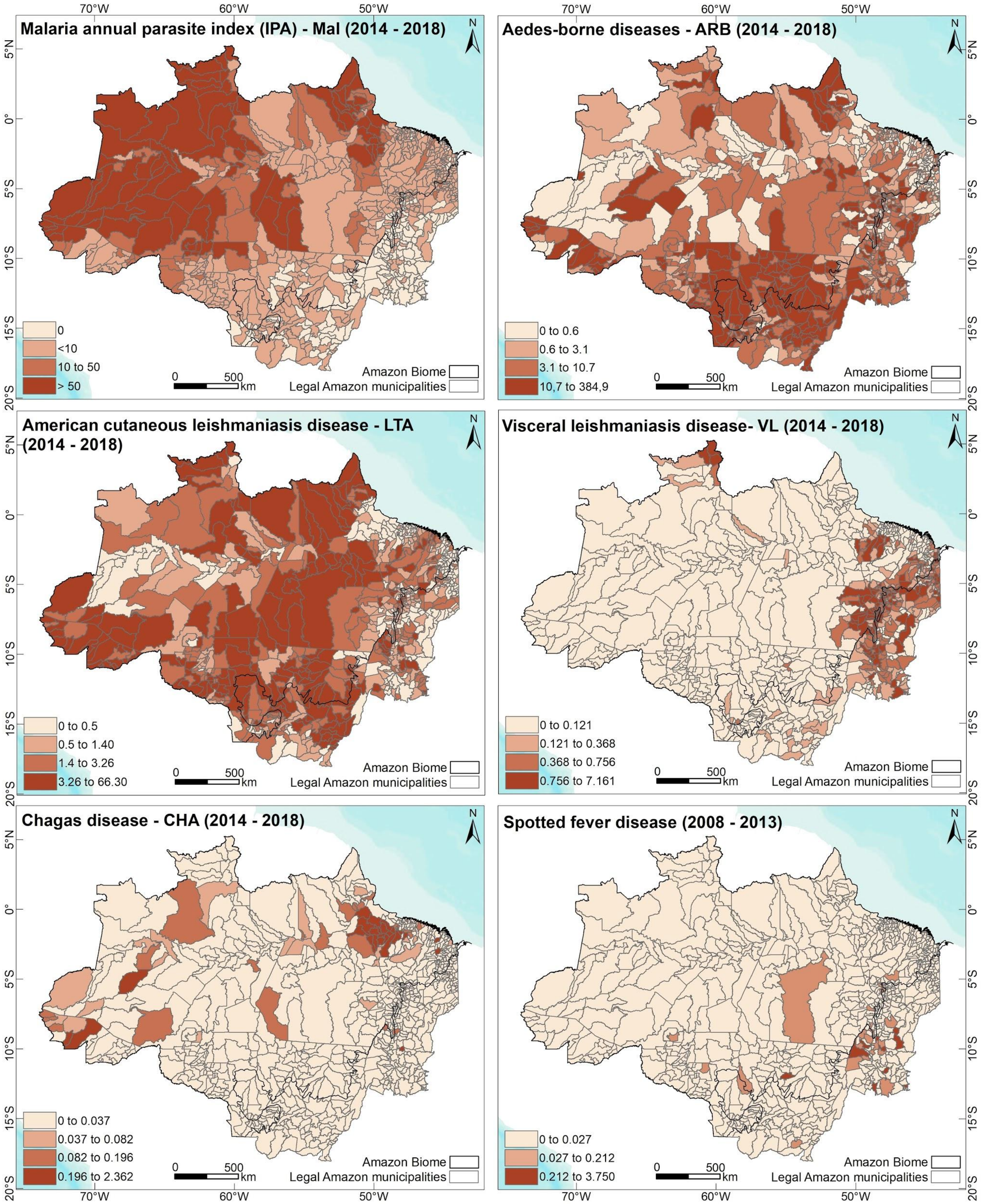

Figure S1. Incidence maps of vector-borne diseases.

Figure S2 shows the 5 years accumulated incidence of 3 non vector-borne zoonotic diseases in the Amazon region.

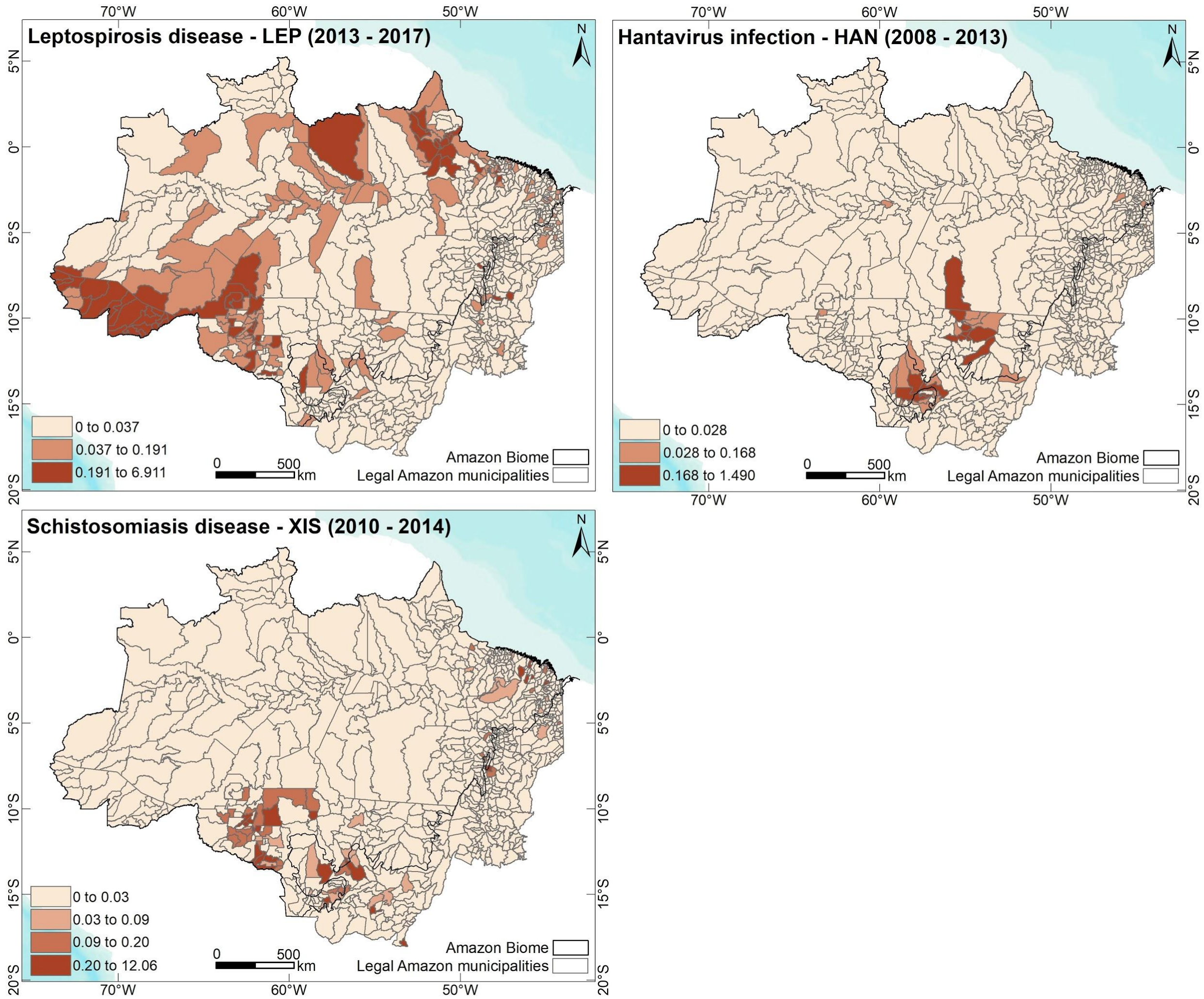

Figure S2. Incidence maps of environment-borne diseases

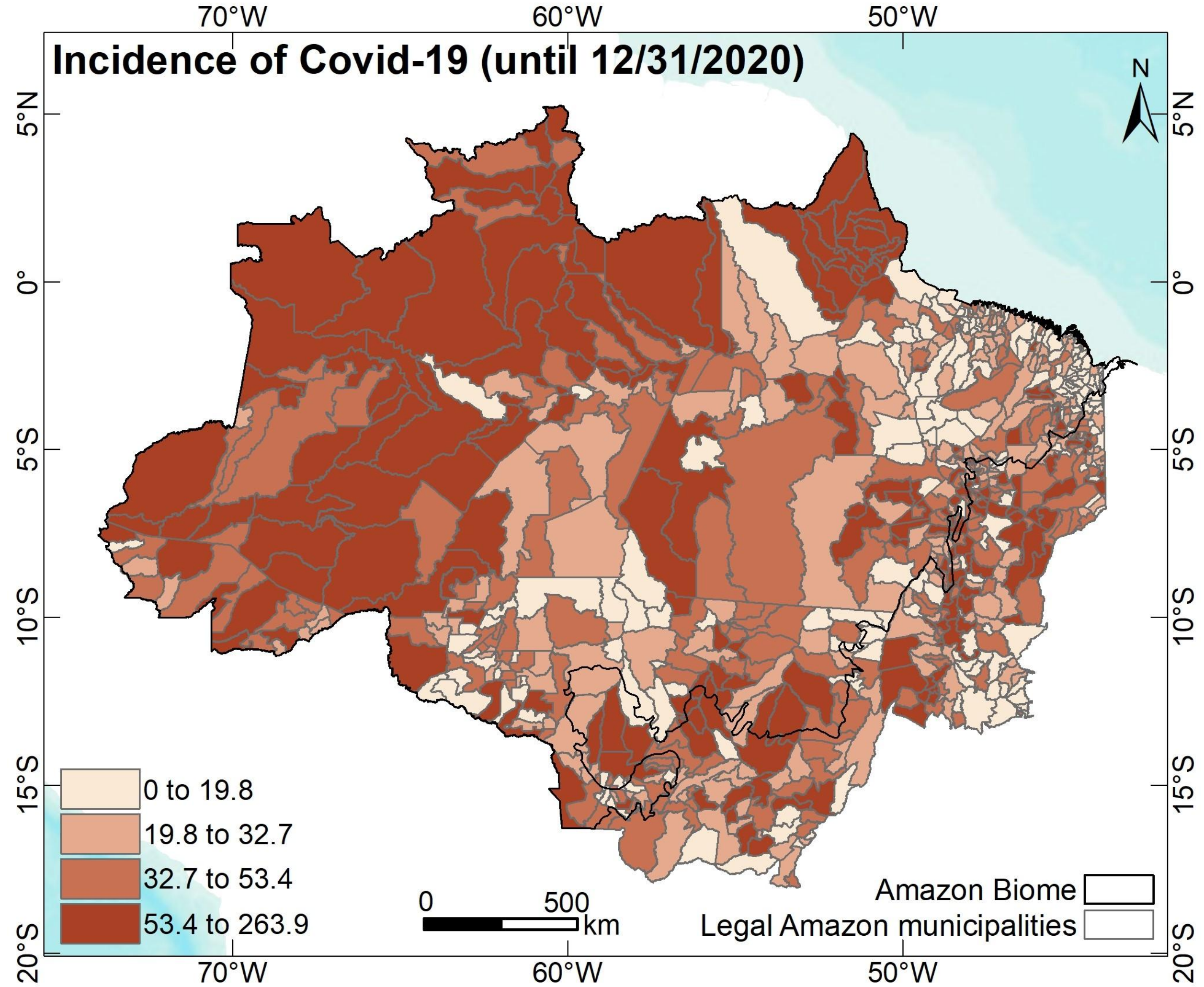

Figure S3. Accumulated incidence of COVID-19 in the Amazon region in 2020.

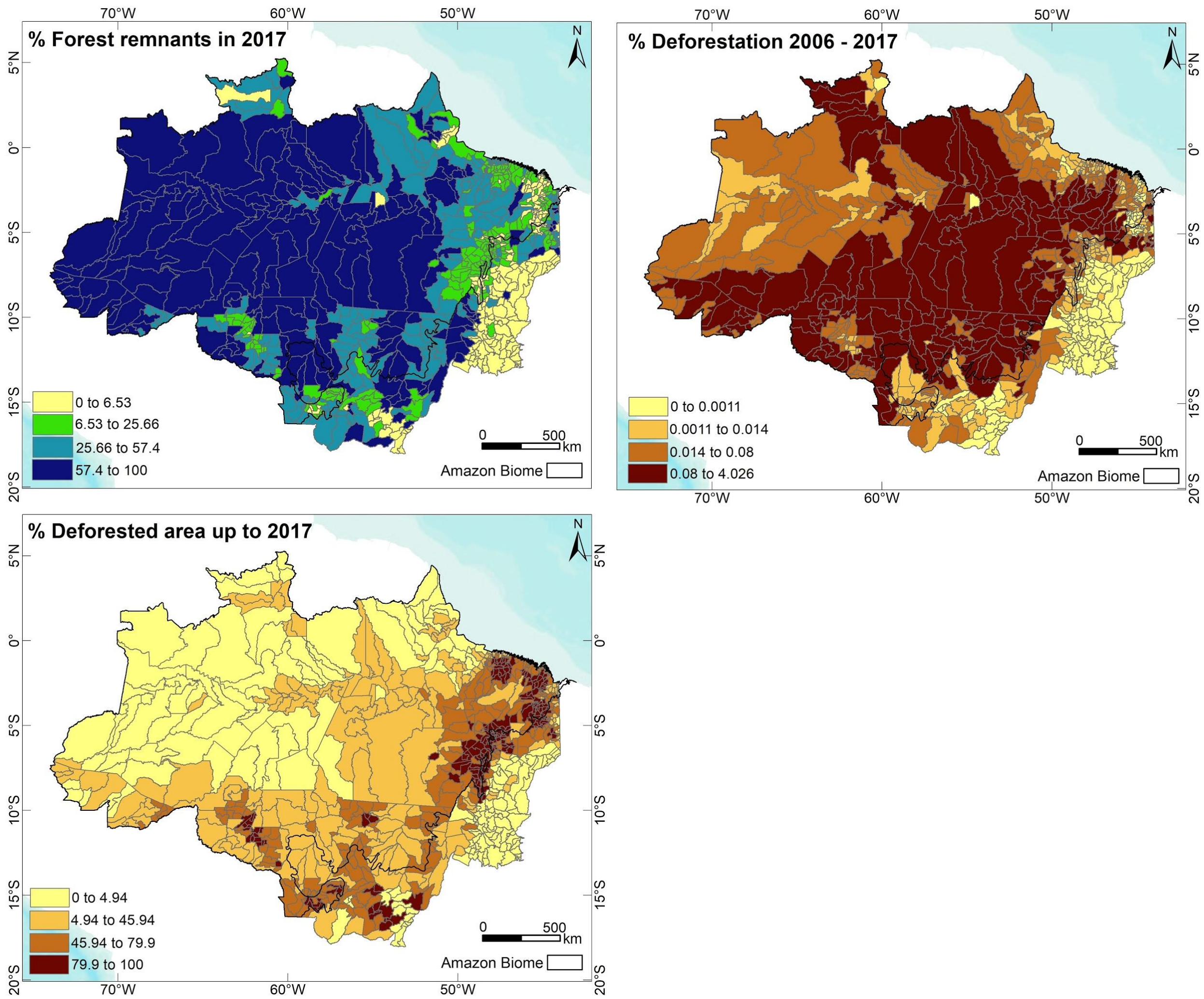

Figure S4. Maps of deforestation indices in the Brazilian Amazon.

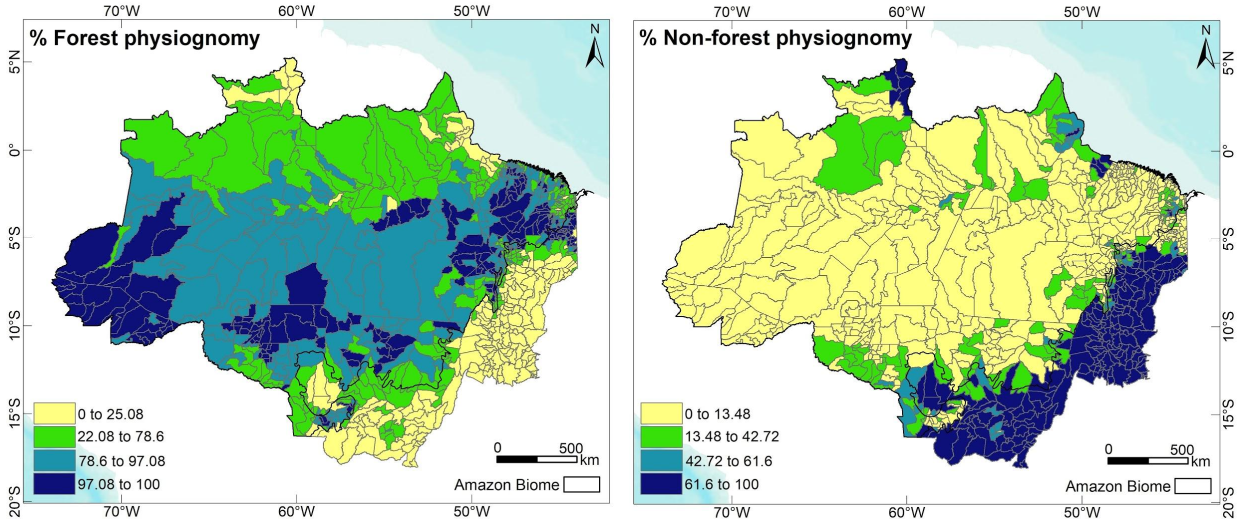

Figure S5. Original physiognomy of the Amazon region.

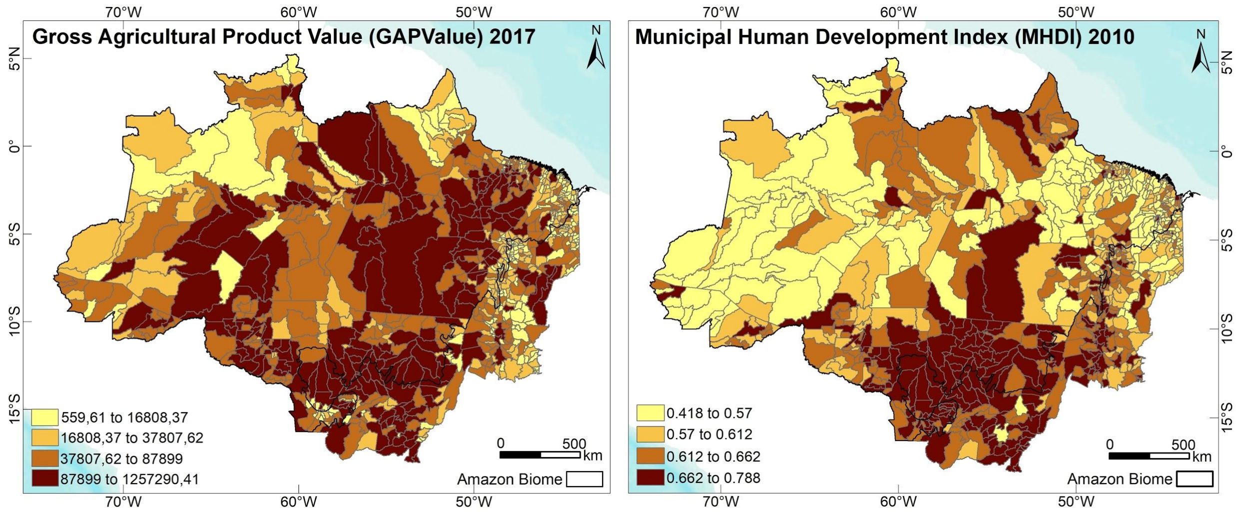

Figure S6. Distribution of GAPValue and MHDI indices in the Amazon region.
